# Untargeted LC-HRMS-based metabolomics to identify novel biomarkers of metastatic colorectal cancer

**DOI:** 10.1038/s41598-019-55952-8

**Published:** 2019-12-27

**Authors:** Ariadna Martín-Blázquez, Caridad Díaz, Encarnación González-Flores, Daniel Franco-Rivas, Cristina Jiménez-Luna, Consolación Melguizo, José Prados, Olga Genilloud, Francisca Vicente, Octavio Caba, José Pérez del Palacio

**Affiliations:** 1 Fundación MEDINA, Centro de Excelencia en Investigación de Medicamentos Innovadores en Andalucía, Granada, Spain; 2 Service of Medical Oncology, University Hospital Virgen de las Nieves of Granada, Granada, Spain; 3 Institute of Biopathology and Regenerative Medicine (IBIMER), Center of Biomedical Research (CIBM), University of Granada, Granada, Spain; 4 Biosanitary Institute of Granada (ibs. GRANADA), SAS-Universidad de Granada, Granada, Spain; 5 Department of Anatomy and Embryology, University of Granada, Granada, Spain

**Keywords:** Oncology, Diagnostic markers

## Abstract

Colorectal cancer is one of the main causes of cancer death worldwide, and novel biomarkers are urgently needed for its early diagnosis and treatment. The utilization of metabolomics to identify and quantify metabolites in body fluids may allow the detection of changes in their concentrations that could serve as diagnostic markers for colorectal cancer and may also represent new therapeutic targets. Metabolomics generates a pathophysiological ‘fingerprint’ that is unique to each individual. The purpose of our study was to identify a differential metabolomic signature for metastatic colorectal cancer. Serum samples from 60 healthy controls and 65 patients with metastatic colorectal cancer were studied by liquid chromatography coupled to high-resolution mass spectrometry in an untargeted metabolomic approach. Multivariate analysis revealed a separation between patients with metastatic colorectal cancer and healthy controls, who significantly differed in serum concentrations of one endocannabinoid, two glycerophospholipids, and two sphingolipids. These findings demonstrate that metabolomics using liquid-chromatography coupled to high-resolution mass spectrometry offers a potent diagnostic tool for metastatic colorectal cancer.

## Introduction

Colorectal cancer (CRC) is still one of the main causes of cancer death^1^. Its pathogenesis has been well characterized, and its development follows a sequence of stages from the transformation of normal colonic epithelium to an adenomatous intermediate polyp and then to an adenocarcinoma^2,3^. Tumor progression involves multiple genetic and epigenetic events, alongside environmental factors. Re-programming of cellular energy metabolism to support continuous cell growth and proliferation has also emerged as a hallmark of CRC^4^. In addition, numerous metabolic enzymes are known to be altered when oncogenes (e.g. RAS, PTEN) or oncogenic transcription factors (e.g. p53, HIF) are affected by mutations. These altered genes produce a chain of metabolic events that can affect glycolytic flux, ATP sources, Krebs cycle and lipid metabolism, among others^5^, and there is abundant evidence of a strong association between lipid metabolism and CRC^6^.

Screening for CRC is essential to reduce its incidence and mortality, but the most reliable method, colonoscopy, is not only invasive but also requires highly specialized practitioners, expensive instrumentation, and specific installations; therefore, it is not always available in settings with limited resources^7^. Intense efforts are warranted to identify new biomarkers of CRC that could overcome these drawbacks. Currently, carbohydrate antigen 19-9 (CA-19-9) is the biomarker of choice to predict CRC^8^.

Metabolomics is a powerful tool that assesses concentrations of low-molecular weight molecules in biological matrices and can be used to generate “metabolic fingerprints” of individuals and diseases. The sensitivity and metabolic coverage of this process has been increased by combining metabolomics with high-resolution mass spectrometry^9^. Such studies have demonstrated an altered metabolism^10,11^ in CRC tissues in comparison to normal mucosa, and studies combining proteomics and metabolomics have described a high rate of aerobic glycolysis in CRC^11,12^. Disorders in several metabolic pathways (fatty acid biosynthesis and oxidative, glycolytic, polyamine pathways, etc.) have also been reported in this disease^13^. However, despite this evidence of metabolic aberrations associated with CRC^14^, it has not proven possible to establish a metabolic profile to serve as a biomarker.

The aim of the present study was to identify potential biomarkers of metastatic CRC by using an untargeted metabolomics approach in serum samples from patients with metastatic CRC and healthy controls using reverse-phase liquid chromatography coupled to high-resolution mass spectrometry (LC-HRMS). The data matrix obtained from these samples showed a significant distinction between HC and CRC groups.

## Methods

### Sample collection

The study included blood samples from 65 patients with metastatic CRC (CRC group) and 60 healthy controls (HC group) matched with the CRC group for age and sex; the median age was 59.9 years in the CRC group, formed by 36 men and 29 women, and 56.1 years in the HC group, formed by 29 men and 31 women. All patients were diagnosed with stage IV disease, without resection of the primary tumor, and liver and/or lung metastasis. Determination of overall survival was carried out (Supporting Information Fig. S1). The variation of different parameters regarding patients and sampling (fasting state, time of day of sampling, etc.) was minimize to obtain the maximum reproducibility in our study. In this way, fasting blood samples were drawn from the patients in hospital between 8 am and 9 am, before the initiation of any treatment and after obtaining written informed consent to participation in the study. The study was approved by the ethics committee of the Virgen de las Nieves Hospital (Granada, Spain), and all clinical investigations were conducted according to the principles expressed in the Declaration of Helsinki (“Ethical Principles for Medical Research Involving Human Subjects”). Samples were collected in BD vacutainer SSTII advance tubes (Becton Dickinson, Franklin Lakes, NJ) with silica to activate clotting of the specimen. After centrifugation for 10 min at 2450 rpm, the supernatant was aspirated and immediately stored at −80 °C until analysis. Serum samples from the healthy controls were supplied by the Biobank of the Andalusian Public Health System and were obtained and treated in the same manner as the samples from patients.

### Metabolite extraction

Serum samples were kept at 4 °C throughout their analysis. First, proteins were removed using acetonitrile (AcN) (1:8 sample/AcN) and agitating for 2 min, followed by centrifugation at 15200 rpm for 10 min at 4 °C. The supernatant was then transferred to HPLC analytical vials and evaporated in a GeneVac HT-8 evaporator (Savant, Holbrook, NY). Dry residues were reconstituted in AcN/water [50:50] with 0.1% formic acid followed by agitation for 1 min.

### LC-HRMS analysis

Extracted serum samples were analyzed using an Agilent 1290 LC system coupled to a Q-TOF 5600 (Triple Quadrupole Time-of-Flight) mass spectrometer (AB SCIEX, Concord, ON) using electrospray ionization in positive mode. Chromatographic separation was performed with an Atlantis T3 HPLC column (C18: 2.1 mm × 150 mm, 3 μm) (Waters Corporation, Milford, MA) kept at 25 °C, injecting 5 μL of sample. The mobile phase was 0.1% formic acid-90:10 water/AcN (eluent A) and 0.1% formic acid-90:10 AcN/water (eluent B). The elution gradient was: 0.00–0.50 min 1% eluent B, 0.50–11.00 min 99% eluent B, 11.00–15.50 min 99% eluent B, 15.50–15.60 min 1% eluent B, and 15.60–20.00 min 1% eluent B. The flow rate was 300 µL/min. The Q-TOF 5600 was operated using a TOF method, providing mass selection with high resolution in combination with information dependent acquisition (IDA), allowing fragmentation of the eight most intense ions and obtaining simultaneously full-scan HRMS and MS/MS data. Ion Source parameters and IDA conditions were: gas source 1: 50.00; gas source 2: 50.00; curtain gas: 45.00; temperature: 500.00 °C; ionspray voltage floating: 4500.00; TOF masses: Min = 80.0000 Da Max = 1600.0000 Da; accumulation time: 0.2500 sec, IDA accumulation time: 0.1000 sec.

A precise mass calibration was performed automatically every 10 injections. Organic solvent (OS) samples and quality control (QC) samples were analyzed during the run sequence. QC samples were prepared by pooling an equal volume of all serum samples used in this study and were run every 10 injections to evaluate the stability and performance of the system. OS samples were run alongside QC samples to identify any impurities from the organic solvents or extraction procedure and to rule out carryover contamination.

### Data set creation

PeakView software (version 1.0 with Formula Finder plug-in version 1.0, AB SCIEX, Concord, ON) was used to evaluate the retention time (RT) and mass/charge (*m/z*) variability of the experiment. MarkerView software (version 1.2.1, AB SCIEX, Concord, ON) was used to process raw LC-HRMS data, carrying out peak detection, alignment, and data filtering and determining the *m/z* ratio, RT, and ion peak area for each sample. Data mining was performed by the program algorithm in the RT range of 1–18 min, and the peak intensity cutoff was set at 200 cps. Peak alignment was achieved using RT and *m/z* tolerances of 0.10 min and 10 ppm, respectively. A filter by “presence” was used to only retain masses appearing in at least 10 samples within the study groups. Next, monoisotopic peaks alone were considered to reduce mass redundancy and enhance selection of a true molecular feature. Finally, mass signals differentially expressed by the OS and case study samples (HC and CRC) were identified by applying an additional filtering procedure with fold change (<1.5) and *t* test (p > 0.05). This procedure eliminated the background and contaminants and preserved the true biological mass signals from LC-HRMS data. The following steps were carried out using the Metaboanalyst 3.0 Web Server.

### Data pre-treatment

Normalization was performed using total area sums, mean centering scaling, and log transformation to transform the data matrix into a more Gaussian-type distribution.

### Analytical validation and outlier detection

The quality of the analytical system performance was evaluated, and principal component analysis (PCA) was used to detect possible outliers. QC samples were displayed on a PCA plot to assess the stability of the analytical system. Variables were removed from the data matrix if their reproducibility was inadequate (relative standard deviation [RSD] > 30% in QC samples) or if they were observed in <50% of QC samples. The goodness of fit (R^2^) and goodness of prediction (Q^2^) of the model were tested. In parallel, one outlier in CRC and four in HC were identified from Hotelling T2 ellipses in partial least squares-discriminant analysis (PLS-DA) despite attempts to modify the standardization method (data not shown). The elimination of outliers did not produce an increase in R^2^ or Q^2^ values.

### Statistical analysis

The survival time of patients was determined by constructing a Kaplan-Meier curve using SPSS 19.0 software. The Student’s *t-*test was used to compare between HC and CRC groups, followed by application of the Benjamini-Hochberg false discovery rate (FDR) correction for multiple comparisons to minimize false positives. An FDR-corrected *p* value of 0.05 was considered the cutoff threshold for the *t-*test. Multivariate analysis was then performed to identify variables that discriminated between the group, combining PCA with PLS-DA. Selection of the features with highest discriminatory power was based on their variable importance in projection (VIP) score, which had to be >1, and on their fold change, which had to be <0.6–>1.5.

### Biomarker identification

PeakView software (version 1.0 with Formula Finder plug-in version 1.0, AB SCIEX, Concord, ON) was used to estimate the elemental formula of selected marker compounds from accurate mass, isotopic clustering, and fragmentation patterns. Next, accurate mass queries were performed in compound databases (Metlin, Human Metabolome Database, Lipid Maps, PubChem, ChemSpider), and fragmentation patterns were searched for in spectral databases (MassBank, NIST2014) for structural identification of the molecular formula. The identification of selected candidates was confirmed by comparison with the accurate mass, RT, and fragmentation pattern of authentic analytical standards.

### Metabolite evaluation

The predictive performance of the selected variables was evaluated by calculating the area under the receiver-operating characteristic (ROC) curve. Univariate and multivariate ROC analyses were conducted to evaluate the clinical value of features as biomarkers either individually or in combination.

## Results and Discussion

### LC-HRMS analysis

**Figure 1:**
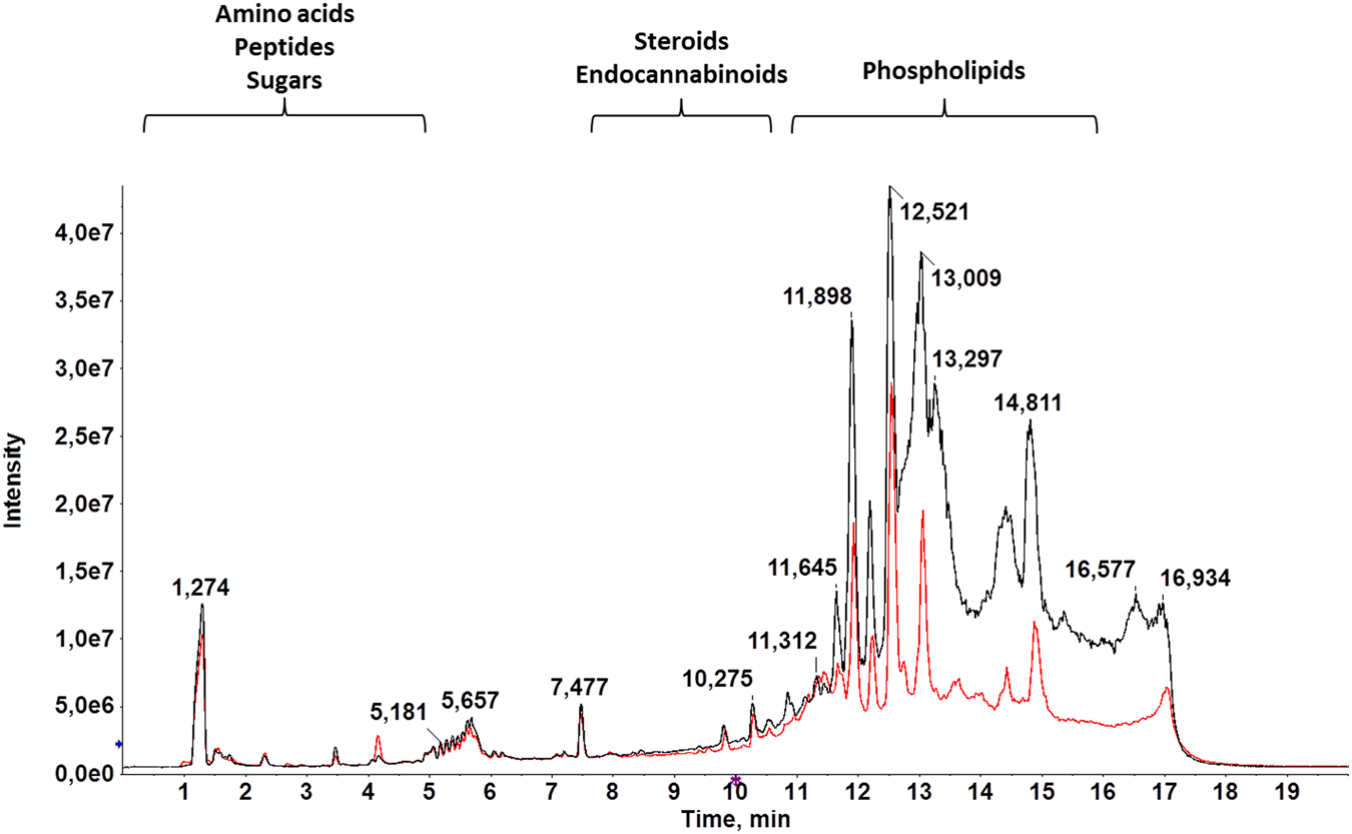
Representative LC-HRMS TIC of the serum sample from a healthy control (HC: black) and patient with CRC (CRC: red). There is a clear separation among: medium-polar metabolites, such as phospholipids, endocannabinoids, or steroids (6–14 min); very polar metabolites, including some amino acids or sugars (1–5 min); and non-polar metabolites. A significant difference between HC and CRC TICs can be observed at 8–15 minutes, when most lipids elute.

AcN was used for extraction in this study, because deproteinization with an OS is a critical step in untargeted metabolomics to avoid ion suppression, and more information about lipid species is obtained in comparison to methanol, the other widely used solvent^15^. LC-MS, selected as analytical platform in this study, has become the technique of choice in metabolomics because of its higher sensitivity and broader range of detectable metabolites in comparison to NMR, the first analytical platform used for the comprehensive measurement of metabolites in a biological sample^16^. The selection of chromatographic mode (reverse phase or HILIC) determines the class of compounds amenable to separation, and a reverse phase column was chosen to achieve the separation of medium-polar and non-polar metabolites^15^. Total ion chromatograms (TIC) of HC and CRC samples (Fig. 1) revealed a clear separation among: medium-polar metabolites (e.g., phospholipids, lysophospholipids, and steroids), eluted between minutes 6 and 14; very polar metabolites (e.g., some amino acids and sugars), eluted in the first 5 minutes and best separated using a HILIC column^17^; and non-polar metabolites, eluted between minutes 14 and 17. According to these findings, the chromatographic process was satisfactory and a significant difference between HC and CRC TICs was observed between minutes 8 and 15, when most lipids elute^18^.

Other researchers have made similar efforts to characterize the metabolomic features of CRC, using different separation techniques and obtaining varying results^19^, but there has been little exploration of potential biomarkers of metastatic CRC. The results of metabolomic studies are influenced by numerous factors, including the sample type, analytical procedure, data treatment workflow, and cohort composition. There is a need to design novel metabolomic approaches to identify new biomarkers that offer an earlier diagnosis of metastatic CRC.

### Chemometric analysis

**Figure 2:**
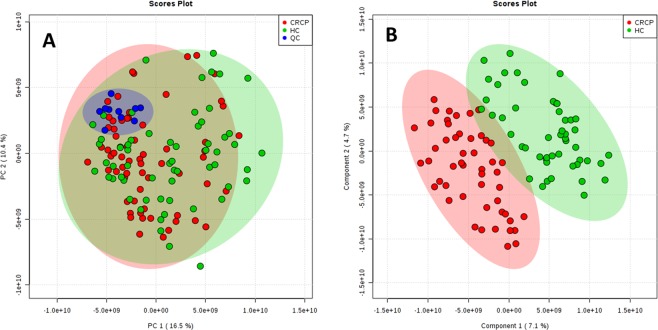
2D score plots of the PCA (**A**) and the PLS-DA (**B**) of the HC group (green) and the CRC group (red). PCA score plot revealed a close clustering of quality control (QC) samples (blue). The PLS-DA score plot suggests that it is possible to discriminate between CRC and HC.

After alignment and filtering procedures, a data matrix of 4301 metabolite features was obtained, including 1868 monoisotopic peaks. Among these features, 1265 were differentially expressed in study samples (HC and CRC) and OS samples. After normalization, 99 features were excluded for unacceptable variability (RSD > 30%); therefore, 1166 variables were evaluated in the PCA. The PCA score plot (Fig. 2A) revealed a close clustering of QC samples, indicating that the separation observed between CRC and HC was mainly due to biological factors. Additionally, the PLS-DA score plot (Fig. 2B) suggested that it might be possible to discriminate between CRC and HC.

The predictive ability of the PLS-DA model to discriminate between CRC and HC was evaluated, adopting the criteria for success of R^2^ ≥ 0.7 and Q^2^ ≥ 0.4, with no variation >0.2–0.3^21^. The present model obtained an R^2^ of 0.74 and Q^2^ of 0.54, demonstrating its suitability for discriminating between HC and CRC samples. Five variables met the criteria established for potential biomarkers of metastatic CRC (FDR-corrected p < 0.05, VIP >1, and fold change >1.5) (Table 1).

**Table 1:** Detailed information on the potential biomarkers of metastatic CRC.

m/z	RT (min)	Molecular formula	Mass error (ppm)	p value	FDR	Fold change*	VIP	AUC	Tentative identification
302.3042^a^	10.12	C18H39NO2	0.5	4.00E-03	2.48E-02	1.81	1.01	0.61	Sphinganine
376.2571^a^	10.89	C24H41NO2	4.2	4.00E-04	4.14E-03	10.68	2.25	0.77	Endocannabinoid
398.2424	10.90	C58H107NO22	1.2	7.00E-04	7.02E-03	6.63	1.86	0.73	Galα1-3(Fucα1-2) Galβ1-4Glcβ-Cer(d18:1/16:0)
500.2724	11.56	C23H44NO7P	0.5	9.43E-10	3.05E-07	0.49	1.12	0.61	PE(18:2(9Z,12Z)/0:0)**
522.3451^a^	11.61	C26H52NO7P	0.6	6.00E-08	2.60E-06	0.58	1.22	0.64	PC (18:1(9Z)/0:0)**

Biomarkers were selected according to t test (FDR correction; p < 0.05), fold change (<0.6–>1.5) and VIP (>1) results. Their potential as clinical biomarkers was evaluated using the area under the receiver-operating characteristic curves. PeakView software was used to estimate molecular formulas. Accurate mass and MS/MS patterns allowed structural identification of the molecular formula.

*Fold change expressed as the ratio of the two averages (HC/CRC).

**PE(18:2(9Z,12Z)/0:0): 1-(9Z,12Z-Octadecadienoyl)-glycero-3-phosphoethanolamine; PC (18:1(9Z)/0:0): 1-(9Z)-Octadecenoyl-sn-glycero-3-phosphocholine.

^a^Confirmed with reference standards.

### Identification of potential biomarkers

**Figure 3:**
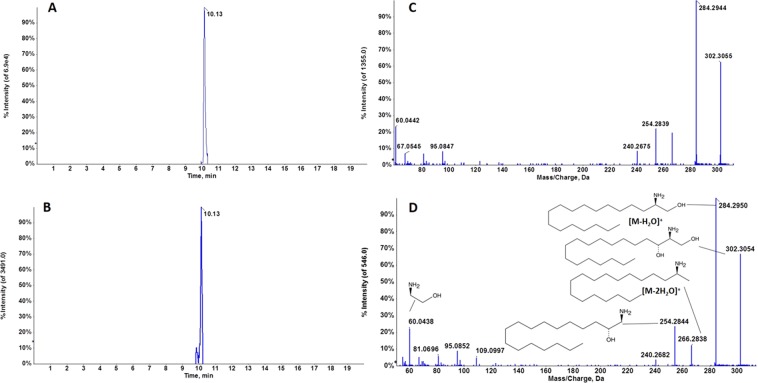
Representative chromatogram of *m/z* 302.3042 in a biological sample (**A**) and sphinganine standard (**B**) at 10.13 min. Characteristic MS/MS spectra of *m/z* 302.3042 in a biological sample (**C**) and sphinganine standard at 10.13 min, and fragment interpretation (**D**). MS/MS spectra reveal the characteristic fragmentation pattern of sphinganine. Fragment ions at *m/z* 284 and 266 have previously been described as a single dehydration and a double dehydration, respectively. It should be noted that the single dehydration product is much more abundant than the double dehydration product, which is also a characteristic pattern.

**Figure 4:**
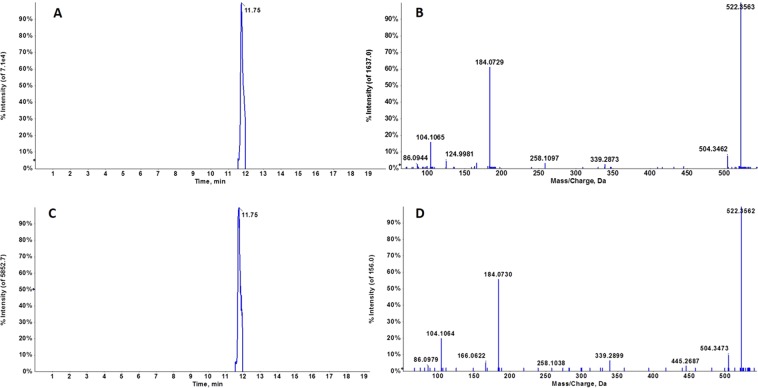
Representative chromatogram of *m/z* 522.3451 in a biological sample (**A**) and PC (18:1) standard (**B**) at 11.75 min. Characteristic MS/MS spectra of *m/z* 522.3451 in a biological sample (**C**) and PC (18:1(9Z)/0:0) standard (**D**) at 11.75 min. Fragment interpretation revealed characteristic ions of phosphatidylcholines, such as *m/z* 104 and 184.

**Figure 5:**
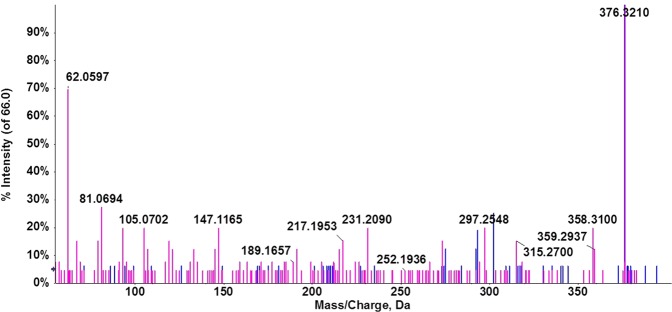
Overlay representation of fragmentation spectra of *m/z* 376.2571 in a biological sample (blue trace) and DEA standard (pink trace).

Queries of accurate mass values in compound databases (Metlin, NIST, LipidMaps and Human Metabolome Database) provided several matches with a mass error below 5 ppm. Evaluation of the molecular formula from accurate mass and isotopic clustering provided a tentative identification of each potential biomarker (Table 1). In addition, interpretation of the experimental fragmentation spectra and comparisons in spectral databases allowed characteristic ions of certain compounds to be recognized. Five compounds were identified in three classes of lipids: sphingolipids (sphinganine, Galα1-3(Fucα1-2)Galβ1-4Glcβ-Cer(d18:1/16:0)), endocannabinoids (docosatetraenoylethanolamide [DEA]), and glycerophospholipids (PE 18:2(9Z,12Z)/0:0) and PC (18:1(9Z)/0:0)). RT and MS/MS spectra of candidate biomarkers were then compared with their corresponding commercial standards in biological samples under the same analytical conditions. The RT and fragmentation pattern were virtually identical between marker and standard in the cases of sphinganine (Fig. 3) and PC (18:1) (Fig. 4)., confirming the tentative identification of these metabolites. A slight difference in RT was observed between DEA and the corresponding standard but the fragmentation patterns were similar, suggesting that this metabolite could belong to the endocannabinoid family (Fig. 5). Unfortunately, no commercial authentic standards of Galα1-3 (Fucα1-2) Galβ1-4Glcβ-Cer (d18:1/16:0) or PE (18:2(9Z,12Z)/0:0 were available, preventing their definitive identification.

### Biomarker evaluation

**Figure 6:**
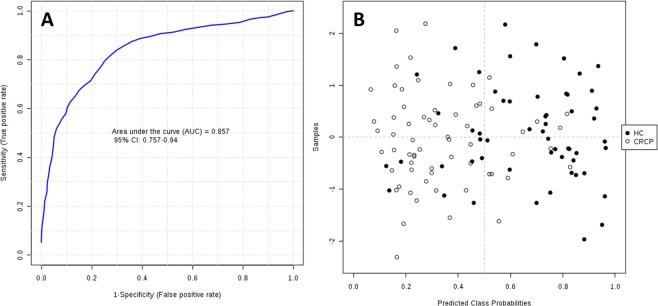
ROC curve for combined biomarker models; 100 cross-validations were performed, and the results were averaged to generate the plot (**A**). Average of predicted class probabilities of each sample in the 100 cross-validations. Because the algorithm uses a balanced subsampling approach, the classification boundary is located at the center (x = 0.5, dotted line) (**B**).

The AUCs obtained in ROC analyses (Table 1) suggest that the five candidate metabolites might potentially serve as biomarkers of metastatic CRC. Following an approach frequently adopted in complex diseases (e.g., cancer or heart or neurological disease), the combination of individual markers in a multivariate model was used to develop a more reliable algorithm. As shown in Fig. 6A, the AUC for the final model was 0.857 (95% CI 0.757–0.940), indicating its strong discriminative power and supporting the value of these metabolites in screening for CRC.

The corresponding confusion matrix (Fig. 6B) shows that 45 HC samples were classified correctly and 15 incorrectly, while 52 CRC samples were classified correctly and 13 incorrectly.

### Biological interpretation

The transformation of a normal cell into a tumor cell mediates through diverse metabolic pathways (e.g., glycolysis, Krebs cycle, urea cycle, osmoregulation, eicosanoid biosynthesis, lipid metabolism, etc.). This altered metabolism might allow us to find several molecules specifically expressed in CRC^20^. In this study, CRC samples revealed alterations in lipid species involved in endocannabinoid, sphingolipid, and glycerophospholipid metabolisms.

Endocannabinoids are known to have many different functionalities in the gastrointestinal tract, specifically in the colon, and they have been reported to inhibit CRC cell proliferation, mainly *via* CB1 receptors^21^. In the present study, endocannabinoid levels were lower in CRC samples than in HC samples, which may be explained by the overexpression in CRC of lipid metabolizing enzyme MAGL, a key enzyme in endocannabinoid metabolism. In this regard, MAGL knockdown models were found to have reduced tumor growth through cyclin D1 and/or Bcl-2 downregulation^22^.

Significant alterations were also observed in two sphingolipids: sphinganine and a neutral glycosphingolipid. Both are N-acylated by ceramide synthases (CerS)^23^ and have shown increased concentrations in CRC^24,25^. It has been reported that human CRC cell apoptosis is promoted by CerS inhibition or the application of ceramide analogues^26^. We therefore hypothesized that decreased sphinganine concentrations might be explained by increased CerS concentrations. Because neutral glycosphingolipids are produced from sphingoid bases, their concentrations are also decreased in CRC^27^. Sphingolipids, key components of biologic membranes, are involved in numerous processes related to tumor progression^23,28^ and have been found to inhibit the growth of cancer cells and induce their apoptosis^29^. Specifically, sphinganine was reported to induce apoptosis in colon cancer cells by arresting the cell cycle at G2/M phase^30,31^. Furthermore, this class of lipids is mediated in part by modulation of the WNT/β-catenin pathway, one of the most important altered pathways in CRC^32^. A downregulation of the aforementioned pathways would result from low concentrations of these sphingolipids, favoring metastasization. The interplay between ceramides and endocannabinoids also appears to be crucial for cancer progression, given that cannabinoids regulate sphingolipid metabolic pathways by promoting sphingomyelin depletion and markedly increasing ceramide concentrations.

Finally, dysregulated choline metabolism has been associated with oncogenesis and tumor progression^33^. Various enzymes are involved in this alteration of choline pathways, including glycerophosphocholine phosphodiesterases (GDPDs)^34^. These enzymes cleave glycerophosphocholine (GPC) to form glycerol-3-phosphate and choline, and their overexpression has been described in cancer, driving tumor cell migration and metastasis^35–37^. In addition, the silencing of GDPDs was found to increase GPC and PE concentrations^38^. Accordingly, increased levels of GDPDs in metastatic CRC would be associated with decreased levels of GPC and PE. Significantly altered GPC and PE species have previously been described in CRC^6^.

We highlight that none of the markers identified in this study have previously been related to CRC^39^ with the exception of LPC (18:1). Interestingly, a significant decrease in LPC (18:1) was observed in non-advanced CRC patients^40^ but an increase in our patients with metastatic CRC, suggesting that this metabolite may potentially be useful as a biomarker of the progression of this disease.

## Conclusions

In this study, LC-HRMS untargeted metabolomics was used to identify a molecular signature that discriminates between individuals with and without CRC, illustrating the potential of this approach for discovering biomarkers. These results contribute new insights into the molecular mechanism and signaling pathways of metastatic CRC and identify novel biomarkers of potential clinical relevance. Further research in larger studies and external validation is warranted to determine the clinical applicability of these metabolic biomarkers in the diagnosis of CRC.

## Supplementary information

Supplementary information 
