# Supplementary material for: Untargeted LC-HRMS-based metabolomics to identify novel biomarkers of metastatic colorectal cancer
Source: Sci Rep. 2019 Dec 27;9:20198. doi: 10.1038/s41598-019-55952-8 (PMC6934557; doi:10.1038/s41598-019-55952-8)
Supplement: Supplementary file 1 — Supplementary information [file 41598_2019_55952_MOESM1_ESM.docx]

**Supplementary information**

**Untargeted LC-HRMS-based metabolomics to identify novel biomarkers of metastatic colorectal cancer**

**Ariadna Martín-Blázquez**^1,+^, **Caridad Díaz**^1,+^, **Encarnación González-Flores**^2^, **Daniel Franco-Rivas**^1^, **Cristina Jiménez-Luna**^3^, **Consolación Melguizo**^3,4,5^, **José Prados**^3,4,5,*^, **Olga Genilloud**^1^, **Francisca Vicente**^1^, **Octavio Caba**^3,4,5,++^ & **José Pérez del Palacio**^1,++^

^1^ Fundación MEDINA, Centro de Excelencia en Investigación de Medicamentos Innovadores en Andalucía, Granada, Spain

^2^ Service of Medical Oncology, University Hospital Virgen de las Nieves of Granada, Granada, Spain

^3^ Institute of Biopathology and Regenerative Medicine (IBIMER), Center of Biomedical Research (CIBM), University of Granada, Granada, Spain

^4^ Biosanitary Institute of Granada (ibs. GRANADA), SAS-Universidad de Granada, Granada, Spain

^5^ Department of Anatomy and Embryology, University of Granada, Granada, Spain

^*^Corresponding author: Jose Prados, jcprados@ugr.es


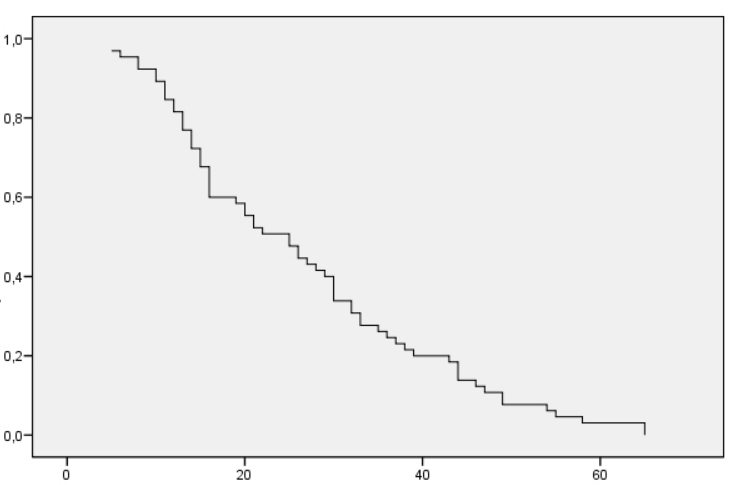


**Overall survival**

**probability**

**Time (months)**

**Figure S1**. Kaplan-Meier survival curve for all metastatic CRC patients in the cohort (n=65).
